# Preliteracy signatures of poor-reading abilities in resting-state EEG

**DOI:** 10.3389/fnhum.2014.00735

**Published:** 2014-09-19

**Authors:** Giuseppina Schiavone, Klaus Linkenkaer-Hansen, Natasha M. Maurits, Anna Plakas, Ben A. M. Maassen, Huibert D. Mansvelder, Aryan van der Leij, Titia L. van Zuijen

**Affiliations:** ^1^Department of Integrative Neurophysiology, Center for Neurogenomics and Cognitive Research, VU University AmsterdamAmsterdam, Netherlands; ^2^Body Area Network, imec/Holst CentreEindhoven, Netherlands; ^3^Department of Neurology, University Medical Center Groningen, University of GroningenGroningen, Netherlands; ^4^Research Institute of Child Development and Education, University of AmsterdamAmsterdam, Netherlands; ^5^Center for Language and Cognition Groningen and University Medical Center Groningen, University of GroningenGroningen, Netherlands

**Keywords:** precursors of reading disabilities, resting-state EEG, reading fluency, delta and alpha oscillations

## Abstract

The hereditary character of dyslexia suggests the presence of putative underlying neural anomalies already in preliterate age. Here, we investigated whether early neurophysiological correlates of future reading difficulties—a hallmark of dyslexia—could be identified in the resting-state EEG of preliterate children. The children in this study were recruited at birth and classified on the basis of parents' performance on reading tests to be at-risk of becoming poor readers (*n* = 48) or not (*n* = 14). Eyes-open rest EEG was measured at the age of 3 years, and the at-risk children were divided into fluent readers (*n* = 24) and non-fluent readers (*n* = 24) after reading assessment at their third grade of school. We found that fluent readers and non-fluent readers differed in normalized spectral amplitude. Non-fluent readers were characterized by lower amplitude in the delta-1 frequency band (0.5–2 Hz) and higher amplitude in the alpha-1 band (6–8 Hz) in multiple scalp regions compared to control and at-risk fluent readers. Interestingly, across groups these EEG biomarkers correlated with several behavioral test scores measured in the third grade. Specifically, the performance on reading fluency, phonological and orthographic tasks and rapid automatized naming task correlated positively with delta-1 and negatively with alpha-1. Together, our results suggest that combining family-risk status, neurophysiological testing and behavioral test scores in a longitudinal setting may help uncover physiological mechanisms implicated with neurodevelopmental disorders such as the predisposition to reading disabilities.

## Introduction

Dyslexia is a learning disorder that specifically impairs a child's technical reading ability. It affects about 5–10% of all children, with higher prevalence in families with one or more members having dyslexia (Lyon et al., 2003). Dyslexia cannot be explained by low intelligence, low-level vision, hearing impairments, or poor education, but phonological deficits and problems with rapid automatized naming are commonly observed reading-related deficits (Snowling et al., 2003; van Bergen et al., 2012). These reading impairments affect the ability to read fluently and are often a cause of frustration and distress for a child, producing severe social and psychological consequences in their lifespan (Vellutino and Scanlon, 1987; Humphrey and Mullins, 2004).

Several genes involved in early brain development have been suggested to cause susceptibility to dyslexia (Scerri and Schulte-Korne, 2010). Thus, even though dyslexia manifests in school years, underlying neural anomalies may already be present in the preliterate brain. This is supported by a number of longitudinal studies using auditory event-related potentials (Molfese, 2000; Guttorm et al., 2001; Maurer et al., 2003, 2009; Lyytinen et al., 2004; Van Zuijen et al., 2012, 2013) and visual event-related potentials (Regtvoort et al., 2006; Schulte-Korne and Bruder, 2010; Araujo et al., 2012) reporting differences in brain responses to reading-related stimuli (e.g., speech sounds or visual contrast) between familial risk and control groups, or between control children and children that later become poor readers.

In adults or school-aged children differences between dyslexics and typical readers have been reported in ongoing EEG activity. Comparing these two groups has pointed to higher delta and theta activity during phonological task (Rippon, 2000; Klimesch et al., 2001a; Spironelli et al., 2006; Penolazzi et al., 2008) and lower alpha and beta activity (Rumsey et al., 1989; Rippon, 2000; Klimesch et al., 2001b) during reading tasks in dyslexics. Several studies (Sklar et al., 1972; Colon et al., 1979; Ahn et al., 1980; Duffy et al., 1980; Pinkerton et al., 1989; Rumsey et al., 1989; Harmony et al., 1995; Clarke et al., 2002; Benasich et al., 2008; Gou et al., 2011; Babiloni et al., 2012) have also investigated EEG from the resting-state. However, differences in participant cohorts—ranging from preschool or school-age children, to adolescents or adults and varying degrees of language disability—and in EEG biomarker definitions has prevented a consensus about possible neuronal signatures of dyslexia.

Throughout development neuronal oscillations plays an important role in shaping the structural and functional neuronal connectivity that will support higher brain functions, such as language and cognition, later in life (Smit et al., 2011). Early impairments in resting-state EEG, reflecting underling neuronal activation, might prelude future developmental problems. In line with these considerations and previous findings, we test whether ongoing neuronal oscillations in preliterate children carry information about reading fluency later in life. We compared relative amplitude spectra of EEG measured at about 3 years of age in three groups of children: one control group of fluent readers and two at-risk groups of fluent and non-fluent readers. Reading fluency was assessed at third grade of school, when the children were about 9 years old. We identified two EEG biomarkers that correlated with reading performance and reading-related test scores collected in third grade.

## Materials and methods

### Subjects and selection criteria

The children that participated in this study are part of the Dutch Dyslexia Programme, a longitudinal research project. The study was approved by the medical ethics committee of the University of Nijmegen, the Netherlands. Informed consent was obtained from one of the parents of each child. Parents were recruited when expecting a baby. The children were first divided in control and at-risk groups, then the at-risk group was divided in fluent and non-fluent readers.

To assess whether the infants were at familial risk of becoming poor readers, the reading fluency of the parents was tested with a word reading task (Brus and Voeten, 1973) and a pseudoword reading task (Van den Bos et al., 1994). In addition, verbal reasoning was measured with the subtest Similarities of the Wechsler Adult Intelligence Scale (Wechsler, 1997; Dutch adaption: Uterwijk, 2000). Children were included in the at-risk group when one of the parents scored (1) lower or at the 20th percentile on both reading tests, (2) lower or at the 10th percentile on one reading test and below the 50th percentile on the other reading test, (3) lower than the 15th percentile on one reading test and not higher than the 40th percentile on the other reading test, or (4) with a discrepancy of 60 percentiles or more between the verbal reasoning test and either of the two reading tests and the additional requirement that both reading scores were below the 50th percentile. The children were included in the control group when both parents scored at the 50th percentile or higher on both reading tests.

Reading in the children was assessed based on three measurements: at the beginning of second grade (2.4 months after starting second grade, SD 1.7 months; the children were 7 years and 6 months of age, SD 5.1 months) with two word-reading lists (1A and 1B from the 3-min test, Verhoeven, 1995), at the end of second grade (6.0 months after starting second grade, SD 0.9 month) with a word reading list (2A from the 3 min test, Verhoeven, 1995) and a pseudoword reading list (Van den Bos et al., 1994), and in the middle of third grade (3.5 months after starting third grade, SD 1.1 month) with a word reading list (Brus and Voeten, 1973) and a pseudoword reading list (Van den Bos et al., 1994). A child was classified as a “non-fluent reader” when it scored poor on two out of three measurements. A child was marked “poor” when it scored below or at the 10th percentile on one of the reading lists and below the 50th percentile on the other reading list, or below or at the 25th percentile on both reading lists. Two children, initially selected as part of the control group, scored as “non-fluent readers” and were excluded from the analysis. One child that was selected to be part of the at-risk group showed general cognitive delay and was omitted as well. This resulted in three groups: a control group of fluent readers (C, 14 children, 9 boys), a group of at-risk fluent readers (RF, 24 children, 16 boys) and a group of at-risk non-fluent readers (RNF, 24 children, 14 boys).

### Behavioral evaluation

Children were submitted to a range of cognitive tests that were administered in the middle of third grade. Two subtests of the Wechsler Intelligence Scale for children (WISC-III, Wechsler, 1992; Dutch adaptation: Kort et al., 2002) were administered: the subtest Block Design measuring nonverbal visual-spatial skills and the subtest Vocabulary measuring expressive vocabulary. Two children from the at-risk fluent group and one child from the at-risk non-fluent group were absent during this evaluation; therefore, they were not included in the behavioral statistical analysis. Behavioral tests for assessing reading-related skills were the rapid automatized naming task (RAN, Van den Bos et al., 1994), a phoneme deletion task (Amsterdamse klankdeletietest, AKT, De Jong and Van der Leij, 2003) and an orthographic choice task (Horsley, 2005). RAN measures the speed of naming overlearned information. The child was requested to name 50 digits from a piece of paper. The naming time was measured and the score was then expressed in the number of digits a child could have named in a minute. The phoneme deletion task measures phonological awareness. The child was asked to repeat a pseudoword (e.g., “memslos”), and subsequently asked to leave out a specific phoneme (e.g., the sound “l”), and to pronounce the resulting word (“memsos”). The score is the number of correct items out of 27. The orthographic choice task measured orthographic knowledge. The child had to decide the correct spelling of a word presented together with two homophonic pseudowords (e.g., among “vurkeer, verkeer, verkir” the correct spelling is “verkeer”). The score was the number of correct answers out of 70 items that the child completed in 10 min.

### EEG recording

Neurophysiological data were collected at 35.1 months of age (SD 0.4 months). EEG was recorded from 64 channels (positioned according to the International 10–20 system; 500 Hz sampling rate; filtered at 0.01–100 Hz), including mastoid references and vertical and horizontal electrooculogram (SynAmps2 64 Channel Quik-Cap, Neuroscan). Three to five min eyes-open rest EEG were collected while the child was on the parent's lap; the child was awake and was encouraged to look at moving lines on a screen to keep it sitting as still as possible. To ensure objectively similar artifact rejection across the different cohorts, the recorded EEG was filtered and cleaned offline using FASTER, a Matlab toolbox for automatic EEG artifacts rejection (for details, see Nolan et al., 2010). In brief, after filtering (0.4–30 Hz band-pass), FASTER segmented the signals into 1-s epochs, detected and interpolated noisy channels, removed contaminated epochs and ran Independent Components Analysis (ICA) for the identification and the rejections of ICs associated with EOG, EMG artifacts. Finally, the signals were re-referenced to the common average, i.e., the average of all remaining scalp electrodes. The mean number of interpolated channels was 2 (± 1 SD, standard deviation) and the mean distance between interpolated channels was in 86% of the cases greater than the distance between neighbor channels. Thus, the influence of the interpolation on the spatial density of scalp EEG was negligible. The duration of the artifact-free epochs in each recording was 1.9 ± 0.1 min (mean ± standard error).

### Spectral analysis of EEG data

Artifact-free epochs were submitted to spectral analysis. Power spectral analysis was computed using Fast Fourier Transform (Welch technique, Hamming windowing function, with 4096 FFT points, resulting in frequency bins of width 0.1 Hz). We observed multiple peaks in the 6–12 Hz range in all subjects and, therefore, we calculated the individual alpha frequency using the gravity frequency peak definition ($\sum \frac{\text{a}\left(\text{f}\right)\times \text{f}}{\sum \text{a}\left(\text{f}\right)}$, with a (f) denoting the power spectral density at frequency f, and ∑ ( ) the sum computed over the frequency bins in the interval 6–12 Hz) (Klimesch, 1999). Mean gravity frequency peak across central electrodes (C1, Cz, C2, CP1, CPz, CP2) was obtained for each subject in each group (mean and standard error of mean for C: 8 ± 0.04 Hz; for RF: 8 ± 0.04 Hz; for RNF: 7.9 ± 0.07 Hz). No statistically significant differences were found between the groups [*F*_(2, 59)_ = 1.17; *p* = 0.3, ANOVA]; similarly, no differences for gender [*F*_(1, 60)_ = 1.06; *p* = 0.3, ANOVA] or for the interaction group × gender were found [*F*_(2, 56)_ = 0.4; *p* = 0.7, ANOVA]. Given these results we considered a common individual alpha frequency of 8 Hz (consistent with literature findings in this age range Stroganova et al., 1999; Marshall et al., 2002) and we defined the frequency bands accordingly (Klimesch, 1999; Babiloni et al., 2012): 0.5–2 Hz (delta-1), 2–4 Hz (delta-2), 4–6 Hz (theta), 6–8 Hz (alpha-1), 8–10 Hz (alpha-2), 10–13 Hz (alpha-3), 13–20 Hz (beta-1), 20–30 Hz (beta-2). The amplitude of EEG signals depends on several factors unrelated to neuro-electrical activity such as anatomical and physical properties of the brain and surrounding tissue (bone thickness, skull resistance and impedance). These parameters vary from one subject to another; however, their influence on the statistical analysis can be minimized by the use of relative amplitude spectra, because these factors equally affect all frequencies analyzed. Amplitude spectra for each electrode were computed as the square root of the power spectra. The relative amplitude spectra were obtained by normalizing as follows:

$$\frac{\langle P_{B_{i}}\rangle }{\sum_{i\;=\;1}^{n}\langle P_{B_{i}}\rangle }$$

where, 〈 〉 indicates the average of the amplitude in a specific frequency band, P_B__i_, across frequency bins, and *i* = 1:n, with *n* = 8, corresponds to the ith frequency band considered in the analysis. The relative amplitude in these eight frequency bands were computed with the NBT toolbox (www.nbtwiki.net) (Hardstone et al., 2012), and are referred to as “biomarkers” following the broad definition: “A characteristic that is objectively measured and evaluated as an indicator of normal biological processes, pathogenic processes, or pharmacological responses to a therapeutic intervention” (Frank and Hargreaves, 2003).

### Statistical analysis for behavioral data

The behavioral tests of word and pseudoword reading, the non-verbal and verbal intelligence subtests, phoneme awareness test, orthographic knowledge test and the RAN tasks were evaluated by One-Way ANOVAs in order to determine differences among the three groups. Tukey's *post-hoc* test was used for *post-hoc* analysis with multiple comparison correction. For correlation analysis with EEG biomarkers, reading fluency performance scores were defined as a composite measure of performance in the two reading tasks measured at the middle of the third grade: word reading and pseudoword reading. Reading fluency performance scores were computed as the z-scores of the average z-scores of the performance in two reading tasks.

### Statistical analysis of EEG data

Distributions of amplitude spectra in each group for each channel and frequency band were tested for normality with the Lilliefors test. For about 25% of the channels the null hypothesis of normal distribution was rejected (*p* < 0.05); for this reason non-parametric methods were used for the statistical analysis. Statistical analysis of differences between groups was performed with non-parametric One-Way ANOVA (Kruskal-Wallis test) with group (C, RF, RNF) as main factor for each electrode and frequency band. Wilcoxon rank-sum test for each channel was used for between-group comparison and to compare means across significant electrodes. As an alternative to multiple comparison correction, we performed binomial testing to validate the statistical significance of the results (Maris and Oostenveld, 2007; Montez et al., 2009; Nikulin et al., 2012); i.e., differences were only considered significant if at least 10 electrodes would reach a *p*-value below 0.05. The likelihood of having this many channels out of 64 reach a *p*-value below 0.05 by chance is less than 0.1% (cf., binomial distribution). No correction was applied across frequency bands.

### Correlation analysis between behavioral and neurophysiological data

The behavioral measures (the reading fluency scores, the scores on phonological awareness, orthographic knowledge, and RAN) that were all assessed in the middle of third grade were correlated with the mean amplitudes across significant channels of the most significant spectral bands using Spearman correlation. Correlation analysis was also performed for each channel as an alternative approach to identify brain regions with activity associated to later reading and reading-related skills.

## Results

### Behavioral results

The three subject groups (control, C; at-risk fluent readers, RF; at-risk non-fluent readers, RNF) were assessed on a variety of reading and reading-related tests (see, Table 1).

**Table 1:** **Behavioral measures**.

**Measure**	**Control**		**Risk fluent**		**Risk non-fluent**		***df***	***F***	***p***
	***M***	***SD***	***M***	***SD***	***M***	***SD***			
**GRADE 2, EARLY**									
RF words 1	64.7_a_	14.9	58.8_a_	16.1	25.0_b_	8.5	(2,57)	51.1	0.001
RF words 2	56.2_a_	21.3	47.0_a_	18.7	14.3_b_	6.4	(2,57)	37.9	0.001
**GRADE 2, LATE**									
RF words	77.5_a_	13.6	61.2_b_	15.9	25.2_c_	9.4	(2,59)	81.5	0.001
RF pseudowords	43.3_a_	13.9	33.6_b_	11.0	15.5_c_	5.5	(2,59)	38.2	0.001
**GRADE 3, MIDDLE**									
RF words	66.5_a_	10.2	56.2_b_	10.6	34.8_c_	7.8	(2,55)	51.7	0.001
RF pseudowords	52.9_a_	14.8	38.0_b_	9.5	20.0_c_	6.6	(2,55)	45.4	0.001
Phon. A.	16.5_a_	1.6	15.6_a_	2.4	11.6_b_	3.8	(2,55)	16.2	0.001
RAN digits	113.6_a_	21.5	109.8_a_	18.5	95.8_b_	12.0	(2,56)	5.8	0.005
Ortho. K.	57.1_a_	8.8	55.3_a_	6.9	35.4_b_	6.7	(2,56)	51.5	0.001
Non-verbal IQ	40.6_a_	13.3	45.9_a_	10.7	42.3_a_	11.3	(2,56)	1.0	0.360
Vocabulary	33.4_ab_	5.7	34.5_b_	3.7	30.3_a_	6.1	(2,56)	3.8	0.027

*Means in the same row that do not share subscript differ in Tukey's post-hoc test at p < 0.05. FR, reading fluency; Phon. A, phonological awareness; RAN, rapid automatized naming; Orhto.K, orthographic knowledge*.

The RNF children scored lower than the two fluent reading groups for phonological awareness (RNF vs. RF, *p* = 0.002; RNF vs. C, *p* = 0.001), RAN digits (RNF vs. RF, *p* = 0.022; RNF vs. C, *p* = 0.01) and orthographic knowledge (RNF vs. RF, *p* = 0.001; RNF vs. C, *p* = 0.001) tasks. This confirms that non-fluent reading is accompanied by reading-related deficits in phonological awareness, rapid naming and orthographic knowledge (De Jong and Van der Leij, 2003; Vellutino et al., 2004).

The groups did not differ on the intelligence subtest of non-verbal IQ; however, the RNF readers scored lower than the RF readers in the vocabulary test (*p* = 0.024). This is a well-known effect and poor verbal abilities have been reported to be present already at preliteracy age in children later diagnosed with dyslexia studies (Snowling et al., 2003; van Bergen et al., 2013).

Performance on word and pseudoword reading fluency tests at the middle of the third grade differed between all groups with the control children performing best, followed by the at-risk fluent readers and the at-risk non-fluent readers (Table 1; for word reading: RNF vs. RF, *p* = 0.001; RNF vs. C, *p* = 0.001; RF vs. C, *p* = 0.007; and for pseudoword reading: RNF vs. RF, *p* = 0.001; RNF vs. C, *p* = 0.001; RF vs. C, *p* = 0.001). This indicates that the RNF children had subclinical reading deficits possibly as a result of a higher liability (van Bergen et al., 2012).

### EEG correlates of future reading abilities

**Figure 1:**
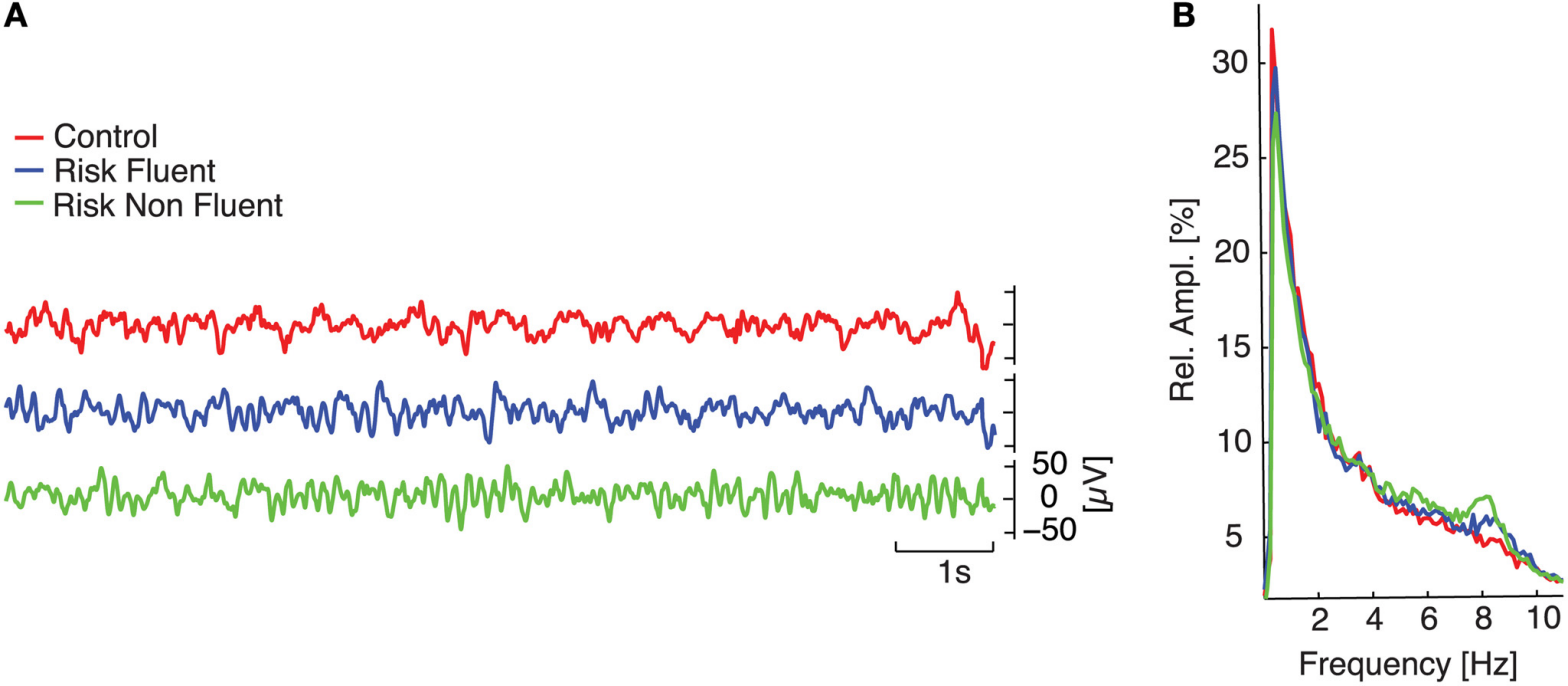
**EEG spectral analysis**. **(A)** temporal evolution of 10 s of filtered signals (0.4–30 Hz) at the central electrode (Cz) for three children belonging to each of the different groups (red line refers to control; blue line to at-risk fluent readers; green line to at-risk non-fluent readers). **(B)** medians of relative amplitude spectra in Cz across each group.

**Figure 2:**
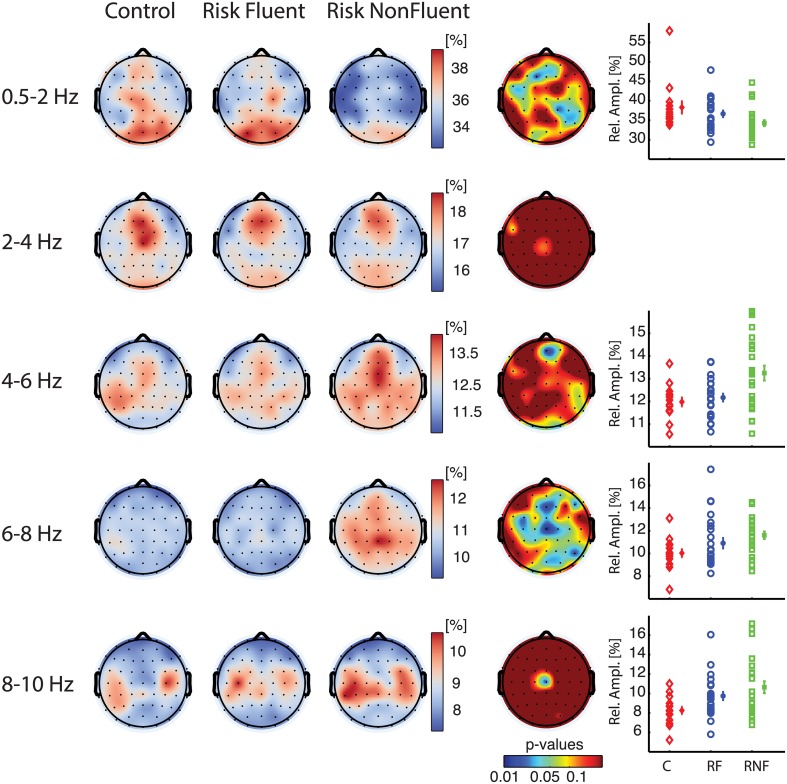
**Relative amplitude spectra in delta-1 (0.5–2 Hz) and alpha-1 (6–8 Hz) bands discriminate children becoming fluent from children becoming non-fluent readers**. Grand-median topographies of delta-1, delta-2, theta, alpha-1, alpha-2 relative amplitude are shown for control (*first column*), at-risk fluent readers (*second column*), at-risk non-fluent readers (*third column*), together with ANOVA *p*-value topographies (*fourth column*). The fifth column contains plots of individual-subject values of mean relative amplitudes across significant electrodes (*p* < 0.05) for each group control (C, red diamonds), at-risk fluent readers (RF, blue circles), at-risk non-fluent readers (RNF, green squares); mean across subjects and standard error bars.

To investigate the presence of differences in preliteracy measures of brain function between those children who would later become fluent readers and those that would become non-fluent readers, we analyzed eyes-open rest EEG recorded at about 3 years of age using classical spectral analysis (Figure 1, and Materials and Methods). We performed an ANOVA to compare relative amplitude in eight different frequency bands among the three groups and observed marked differences in the delta-1 and alpha-1 bands (Figure 2). Electrodes with a significant effect formed spatially connected clusters over frontal and centro-parietal scalp regions for the delta-1 band and over central and parieto-occipital regions for the alpha-1 band (see *p*-value topography maps in Figure 2, fourth column). For delta-1 we found 10 electrodes with a significant difference between groups at *p* < 0.05, whereas 18 electrodes reached this level for alpha-1. According to binomial testing, the probability of obtaining an equal or larger number of electrodes than 10 was negligibly small (*p* < 1e−3; Materials and Methods). On the contrary, for the theta and alpha-2 bands the number of significant electrodes was too small to reject the null hypothesis of the binomial significance test. The ANOVA for delta-2 did not show a main group effect at any electrode. No effects were observed in the higher frequency bands of alpha-3, beta-1, or beta-2 (data not shown). We note that alpha-2 oscillations displayed peaks bilaterally over the sensorimotor regions for all groups as previously reported for this age (Marshall and Meltzoff, 2011), which suggests that the alpha-1 result is not the child equivalent of the sensorimotor mu rhythm.

**Figure 3:**
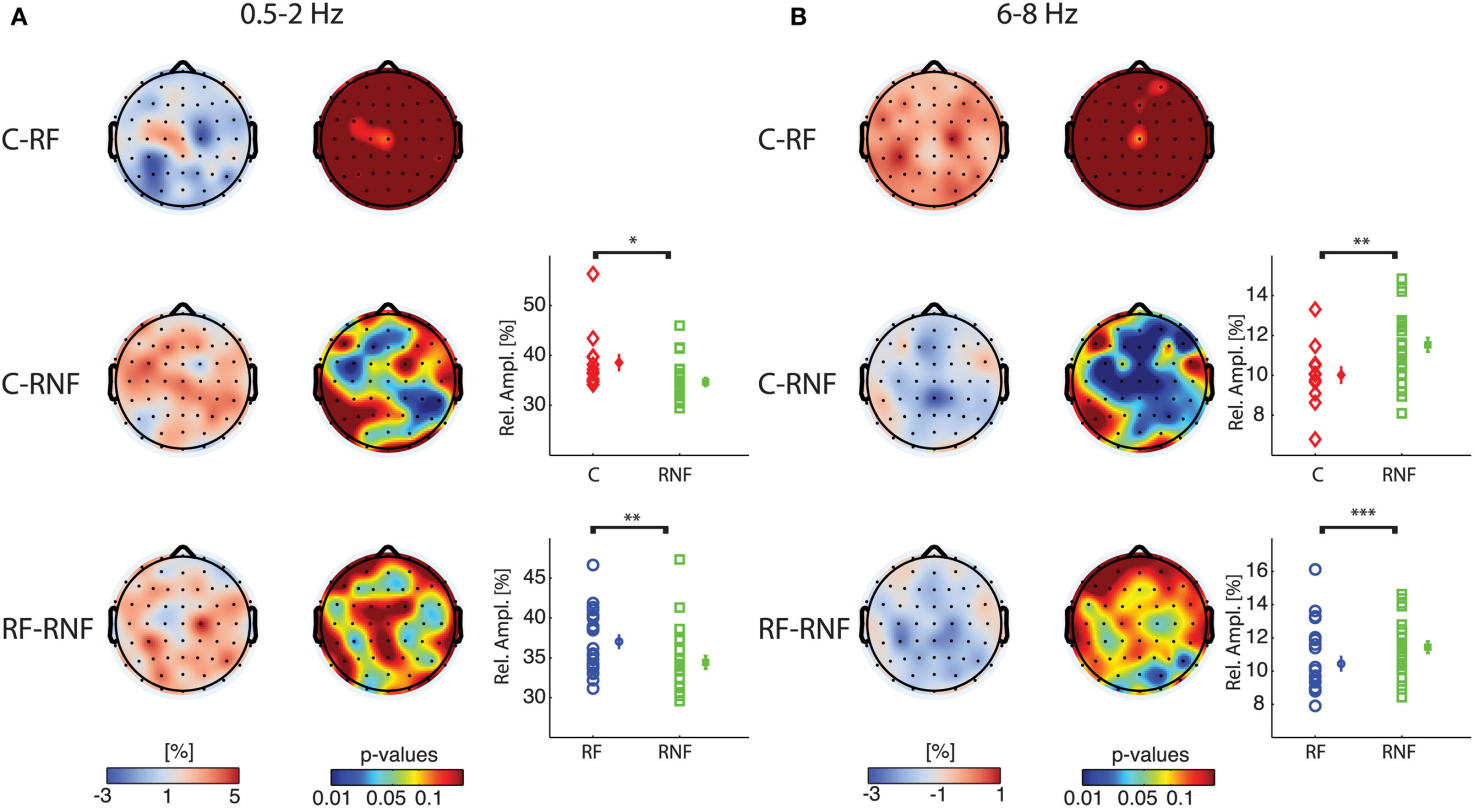
**At-risk non-fluent readers have altered delta-1 and alpha-1 compared to control and at-risk fluent readers**. Pair wise group statistics (rank-sum tests) are displayed for relative amplitude for delta-1 **(A)** and alpha-1 **(B)**. In the first column: topographies of the difference of grand median values (cool-warm colormap) between controls and at-risk fluent readers (C-RF) in the first row, between controls and at-risk non-fluent readers (C-RNF) in the second row, between at-risk fluent readers and at-risk non-fluent readers (RF-RNF) in the third row. In the second column: *p*-value topographies for the three comparisons (jet colormap). In the third column: plots of individual-subject values of mean relative amplitudes across significant electrodes (*p* <0.05) for each group control, mean across subjects and standard error bars (refer to Figure 2 for explanation). The asterisks indicate significant differences obtained with rank-sum test, comparing the individual-subject average across significant electrodes between groups (^*^p = 0.005;^**^p = 0.01; ^***^p = 0.03).

To identify more specifically the group differences that gave rise to the significant effects in the overall group comparisons we performed rank-sum tests between groups for delta-1 and alpha-1 relative amplitude. The results indicated that the control group did not differ from the at-risk fluent group in either of the two frequency bands (Figures 3A,B, first row), whereas widespread differences were observed for the comparisons of the control and at-risk non-fluent groups as well as for at-risk fluent and the at-risk non-fluent groups. Relative delta-1 activity was low in at-risk non-fluent group compared to both the control and at-risk fluent group (Figure 3A, second and third row), whereas relative alpha-1 activity was higher in the at-risk non-fluent group compared to both control and at-risk fluent group (Figure 3B, second and third row). The number of electrodes with *p* < 0.05 in these comparisons varied between 7 and 33; the probability of obtaining an equal or larger number of electrodes for a binomial distribution was small (*p* < 0.03; Materials and Methods). Although one should be cautious in inferring the origin of the sources giving rise to the scalp topographies shown in Figure 3, they could correspond to brain reading circuits identified in several neuroimaging studies to include parieto-temporal, occipito-temporal and inferior frontal lobes (Eckert et al., 2005; Richlan et al., 2009; Raschle et al., 2011). Together, these results indicate that children who became poor readers exhibited already at the age of three a peculiar resting-state EEG activity compared to both control and at-risk fluent groups.

### Correlation between EEG biomarkers and behavioral measures

**Figure 4:**
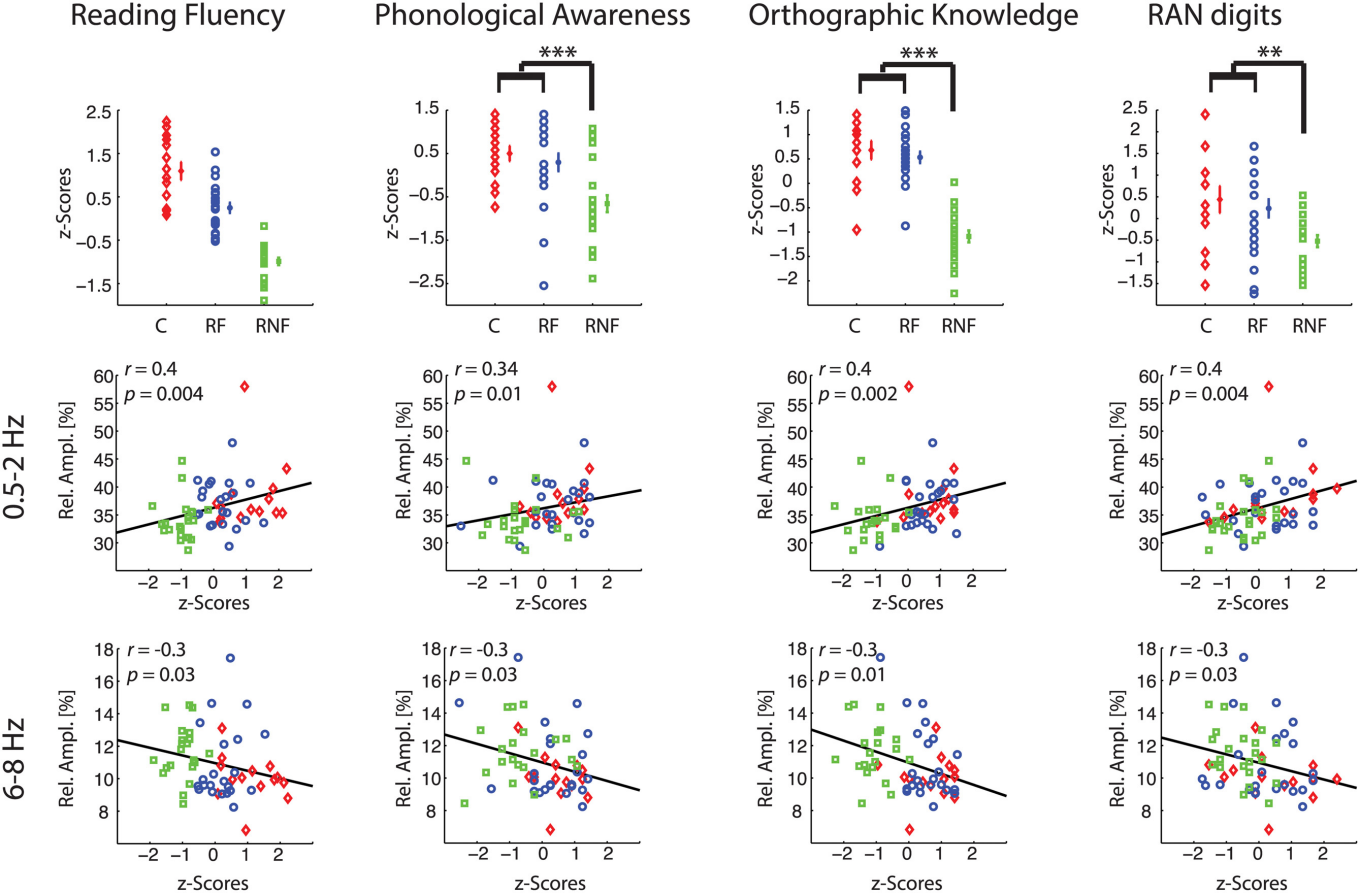
**Correlations between behavioral and neurophysiological measures**. Each column refers to a specific behavioral task. Plots in the *first row* illustrate individual-subject z-scores, across subject means, and standard error bars for each group (control, C, small red diamonds; at-risk fluent readers, RF, blue circles; at-risk non-fluent readers, RNF, green squares). The asterisks indicate *p*-values of the statistics in Table 1 (^**^p = 0.005; ^***^p = 0.001). For the reading fluency assessment (composite measure of performance scores in word reading and pseudoword reading tasks) in the top left plot, all the groups statistically differ (*p* = 0.001). The scatter plots in the remaining rows represent the correlations between individual-subject z-scores at each reading-related test and the individual-subject average across significant electrodes (*p* < 0.05) of relative amplitude in delta-1 (*second row*), and in alpha-1 (*third row*). Statistics is based on *n* = 59 children.

Having identified relative amplitude of delta-1 and alpha-1 as putative biomarkers of future ability to read, we subsequently examined whether these EEG biomarkers could be related to performance in reading fluency and reading-related abilities (Figure 4, first row).

**Figure 5:**
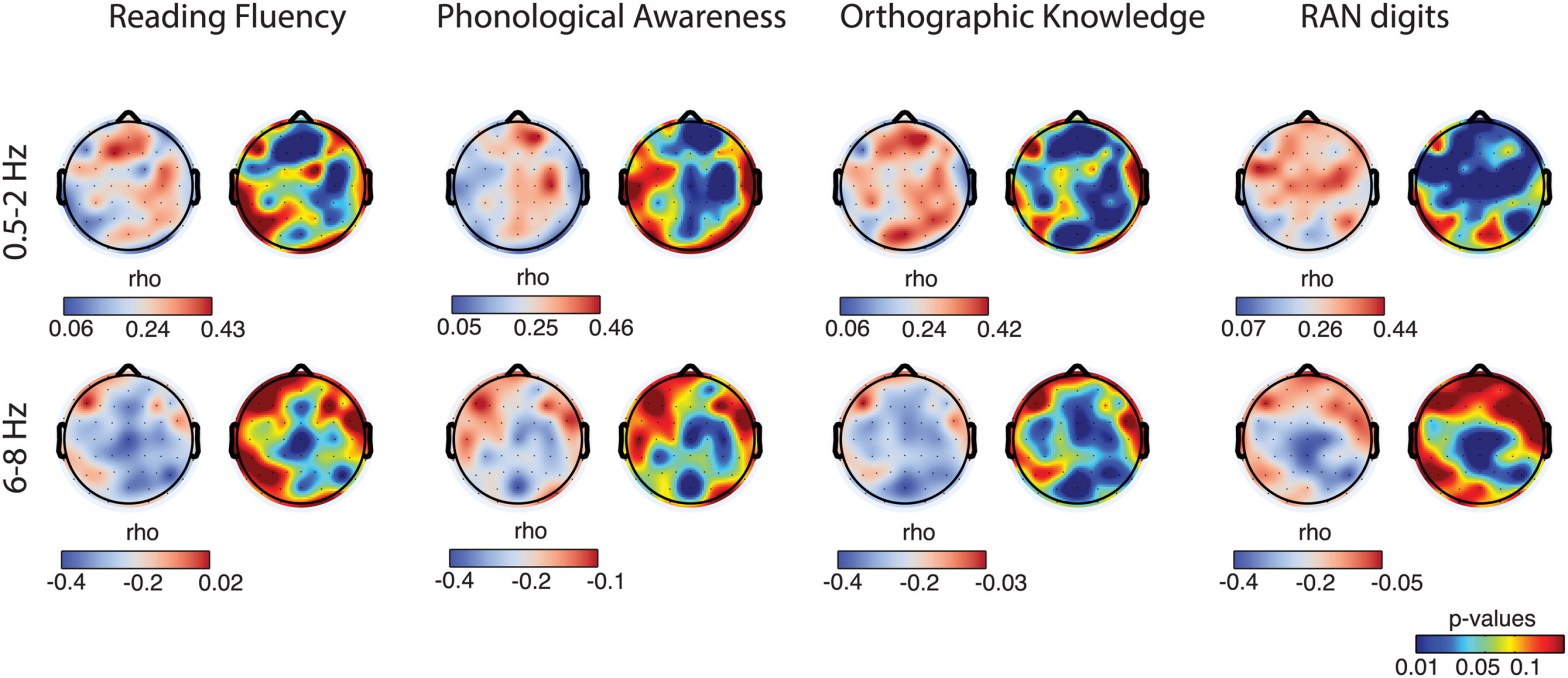
**Correlation topographies**. For each behavioral task, correlations with delta-1 (*first row*) and alpha-1 (*second row*) relative amplitude of all the subjects are shown in the form of correlation coefficient (r) topographies (cool-warm color map) and *p*-value topographies (jet color map).

First, we computed the mean biomarker values of the delta-1 and alpha-1 across significant channels (see Figure 2, fifth column) and correlated these with the z-scores of the performance in the behavioral tests measured in the middle of the third grade. Within-group correlations did not reach statistical significance, which was expected given the restriction of range for the performance scores within groups. On the other hand, when considering all children together significant correlations were found between the EEG biomarkers and all the behavioral tests, i.e., Reading Fluency, Phonological Awareness, Orthographic Knowledge and RAN digits. Increasing performance on the different tasks correlated with increases in delta-1 amplitudes and decreases in alpha-1 amplitudes (correlation coefficients and *p*-values are reported in Figure 4, second row and third row). This result was to some extent expected given the group effects in the EEG and in the behavioral tests. Interestingly, however, when using the full range of correlations, scalp topographies revealed that also channels not showing a significant group difference were found to correlate with future performances (Figure 5).

Scalp topographies of correlations between the behavioral measures and the EEG biomarkers again suggested the involvement of multiple brain regions. In particular, delta-1's relative amplitude correlations with Reading Fluency, with Phonological Awareness and with RAN digits appeared both in frontal, central, and parieto-temporal regions. Similar regions showed correlations with Orthographic Knowledge and, interestingly, also occipito-parietal regions showed a robust positive correlation (Figure 5, first row) with Orthographic Knowledge. For alpha-1's relative amplitude significant correlations were stronger in central sites and correlations with performance on the orthographic task were also observed in the occipital region (Figure 5, second row). Noteworthy, correlations over occipital sites were more evident in the orthographic task where processing of visual information takes place. Additionally, the correlations we have found over frontal and parietal electrodes for the phonological task might be in agreement with previous studies associating phonological processing with activity in frontal lobes and parietal and temporal regions (Buchsbaum, 2001; Burton, 2009).

## Discussion

The present study aimed at investigating neurophysiological correlates of later emerging reading abilities in the preliterate brain. Uniquely to our study, having longitudinal data covering about 9 years of development, we showed that characteristic spectral pattern in the resting-state EEG activity of children at 3 years of age discriminate children that became poor readers from children that became fluent readers. The ability to read fluently develops gradually over time and through substantial practice but in dyslexics it is hampered by the presence of reading-related deficits in phonological processing, mapping phonemes to graphemes, and automatic word recognition. Our behavioral results confirm this effect in poor readers showing that at-risk non-fluent children scored lower than both at-risk fluent and control children in all reading-related tests (Phonological Awareness, Orthographical Knowledge and RAN). Our data on intelligence measures were in agreement with previous findings showing that dyslexics score slightly lower on verbal tasks despite having adequate reasoning abilities compared to non-impaired readers (Snowling et al., 2003; van Bergen et al., 2013).

The comparison of EEG relative amplitude between the three groups of children, divided on the basis of their family-risk status and their reading fluency abilities, revealed several interesting findings. Our results show that two EEG biomarkers, delta-1 and alpha-1 relative amplitudes, emerged as putative preliterate discriminants of those children with a familiar risk of dyslexia who did become non-fluent readers. Non-fluent readers exhibited significantly lower levels of delta-1 activity and significantly higher alpha-1 activity compared to fluent readers (both control and at-risk fluent readers). Noteworthy, although the alpha-1 effect was prominent in central regions, we believe this does not reflect a sensorimotor mu rhythm deficit, because of the larger amplitude and clear bi-lateral topographic distribution of relative amplitude seen in alpha-2 band (Marshall and Meltzoff, 2011). Topographic distributions of the main group effect and of the correlations between the EEG biomarkers and behavioral data suggested the involvement of several scalp regions, which is in line with neuroimaging studies that have identified reading circuits both in parieto-temporal, occipito-temporal and inferior frontal lobes, albeit often with a left laterality (Eckert et al., 2005; Richlan et al., 2009; Raschle et al., 2011). Given that our statistical analysis was performed in sensor space, we are cautious with the interpretation of the source origin of the effects shown in Figures 2, 3, 5; however, we note that both delta-1 and alpha-1 relative amplitude exhibited strong correlations with Orthographic Knowledge in occipital areas, which have previously been associated with orthographic processing (Samuelsson, 2000). Differently, phonological processing is known to involve frontal areas (Burton, 2009), which could explain the prominent effects we have found in the frontal scalp regions, especially for the delta-1. These results might suggest a broader role of delta activity and a specificity of alpha-1 oscillations for visual processing, present even before the reading onset.

### The function of delta and alpha oscillations in brain maturation

It is well known that delta activity dominates the human EEG during early development and decreases over the course of normal development (John et al., 1980; Gasser et al., 1988; Harmony et al., 1990). Slow wave delta activity in development is believed to be important in pruning redundant cortical connections and supports brain maturation as reflected in the positive association between delta activity and gray matter volume (Whitford et al., 2007). If slow-wave delta activity is indeed a necessary mechanism for pruning and cortical development to take place, our finding of reduced delta-1 activity seem to be in agreement with the hypothesis of a cerebral maturation delay in 3-year old children that later on become poor readers. Later in life, delta waves remain dominant during slow-wave sleep; however, a relatively high delta activity in a wakeful state has been associated with pathological neuronal conditions (Spironelli and Angrilli, 2009; Babiloni et al., 2012) such as in adults with ADHD and dyslexia (Chabot et al., 2001; Penolazzi et al., 2008). Higher delta activity has also been observed in dyslexic school-age children (Spironelli et al., 2006; Penolazzi et al., 2008; Spironelli and Angrilli, 2010) and dyslexic young adults (Rippon, 2000), although these data were recorded during reading tasks. However, increased delta and theta activity in dyslexics or children with reading and writing disabilities have also been reported in resting-state EEG at the age of 9–18 years (Sklar et al., 1972; Colon et al., 1979; Pinkerton et al., 1989; Harmony et al., 1995). Thus, in line with the hypothesis of dysfunctional development (Spironelli et al., 2006, 2010, 2011; Penolazzi et al., 2008), it is plausible that failure to produce adequate delta at a young age is part of the mechanism causing a delay of cortical maturation, which in turn may be reflected in an increase of delta activity in at-risk non-fluent readers compared to fluent readers at school-age. To investigate this, future analyses will be done on resting-state EEG collected in the present cohort at the age of 11 years.

In the present study, we used relative amplitude measures to reduce the considerable genetic variance on oscillatory power (Linkenkaer-Hansen et al., 2007). Thus, it is plausible that our findings in the alpha-1 bands are to some extent related to those in the delta band. On the other hand, increases of lower alpha (spectral component just below the IAF) activity have been associated with difficulty in sustaining attention and inhibiting distracting environmental stimuli (Klimesch et al., 2001b). Thus, we cannot rule out that the children in our study differed in the level of attention paid to the moving lines, with the non-fluent reading group exhibiting low sustained attention, as reflected in higher alpha-1 amplitude compare to the other two groups.

### Comparison with other familial risk studies

It is worth noting that our cohort resembles the cohort of the studies presented in Benasich et al. (2008) and Gou et al. (2011) where resting-state EEG of age-matched children (16, 24, and 36 months of age) with a family history of language-based learning impairment (LLI, FH+) and controls (FH−) were compared. Benasich et al. (2008) performed qualitative analysis among absolute power spectra in 9 frequency bins ranging from 5 to 50 Hz (excluding frequency components of the delta range) and across different scalp regions. They selected frontal and prefrontal regions and focused on two wide frequency ranges for their statistical analysis (5–30 Hz and 31–50 Hz, of which the latter was referred to as gamma). They reported lower frontal gamma power in FH+ compared to FH-, and found correlations of gamma power with attention, and expressive and receptive language skills measured at the same age. In a later study, using the same cohort of children, Gou et al. (2011) reported that resting frontal gamma power at 16, 24, and 36 months was associated with phonological memory and syntactical skill measured at the age of 4 and 5 years. Comparison with the present study are not possible for several reasons: (1) the definition of the family risk in our study is based on the parents' performance on reading tests, whereas in Benasich et al. (2008) it is associated to the presence of at least one sibling or parent diagnosed with LLI (75% were siblings Choudhury and Benasich, 2003); (2) none of the children in Gou et al. (2011) were themselves diagnosed with any language or learning disabilities, whereas our at-risk children were divided into groups that became fluent or non-fluent readers; (3) the EEG signal analysis performed in our study did not include frequency components higher than 30 Hz. Despite the differences in the cohort definition and in the EEG analysis, our results are congruent with the findings of Benasich et al. (2008) related to absence of statistical difference between control and at-risk fluent readers (comparable with FH+) for spectral amplitude lower than 30 Hz.

## Conclusions

In conclusion, we showed that combining family-risk status assessment and resting-state EEG at preliterate age could provide preliminary indicators of future reading abilities. Specifically, our data suggest that delta and alpha oscillations are implicated with neurophysiological processes of importance for reading-related disabilities later in childhood. In particular, we confirm the role of delta activity as a physiological index of abnormal cerebral maturation. Further investigations are required to better understand the functional significance and the underlying mechanisms governing the dynamics of these oscillations in developmental dyslexia. For example, in this study we did not account for special training in addition to schooling that some poor-reading children have followed, partially influencing their performances in the reading-related tasks measured at the middle of the third grade. In this regard, future studies may investigate whether reading-intervention programs (Connor et al., 2007)affect the dynamics of the brain as reflected in the resting-state EEG.

### Conflict of interest statement

The authors declare that the research was conducted in the absence of any commercial or financial relationships that could be construed as a potential conflict of interest.
